# Evolutionary emergence of angiogenesis in avascular tumors using a spatial public goods game

**DOI:** 10.1371/journal.pone.0175063

**Published:** 2017-04-11

**Authors:** Javad Salimi Sartakhti, Mohammad Hossein Manshaei, David Basanta, Mehdi Sadeghi

**Affiliations:** 1 Department of Electrical and Computer Engineering, Isfahan University of Technology, Isfahan 84156-83111, Iran; 2 Integrated Mathematical Oncology, H. Lee Moffitt Cancer Center and Research Institute, Tampa, FL 33612, United States of America; 3 National Institute of Genetic Engineering and Biotechnology, Tehran, Iran; 4 School of Biological Sciences, Institute for Research in Fundamental Sciences, Tehran, Iran; Beihang University, CHINA

## Abstract

Natural selection in cancer often results in the emergence of increasingly malignant tumor cells that display many if not all of the hallmarks of cancer. One of the most important traits acquired during cancer progression is angiogenesis. Tumor cells capable of secreting pro-angiogenic factors can be seen as cooperators where the improved oxygenation, nutrient delivery and waste disposal resulting from angiogenesis could be seen as a public good. Under this view, the relatively costly secretion of molecular signals required to orchestrate angiogenesis would be undertaken exclusively by cooperating tumor cells but the benefits of angiogenesis would be felt by neighboring tumor cells regardless of their contribution to the process. In this work we detail a mathematical model to better understand how clones capable of secreting pro-angiogenic factors can emerge in a tumor made of non-cooperative tumor cells. Given the importance of the spatial configuration of the tumor in determining the efficacy of the secretion of pro-angiogenic factors as well as the benefits of angiogenesis we have developed a spatial game theoretic approach where interactions and public good diffusion are described by graphs. The results show that structure of the population affects the evolutionary dynamics of the pro-angiogenic clone. Specifically, when the benefit of angiogenesis is represented by sigmoid function with regards to the number of pro-angiogenic clones then the probability of the coexistence of pro-angiogenic and angiogenesis-neutral clones increases. Our results demonstrate that pro-angiogenic clone equilibrates into clusters that appear from surrounding vascular tissues towards the center of tumor. These clusters appear notably less dense after anti-angiogenic therapy.

## Introduction

It is well known that natural selection drives cancer somatic evolution [1–4]. As they grow, tumors become increasingly heterogeneous and the different clones in the tumor compete for scarce available resources like space, oxygen and glucose. Thus, clones that are better adapted will gain relative proliferative and/or survival benefits [5]. Explaining this behavior and dynamics is easier if we consider that selection in cancer’s somatic evolution results from the interactions between tumor clones with different phenotypes. For instance, a proliferative clone must obtain sufficient resources to sustain increased proliferation. So, this clone can get upper hand in population [5]. However, tumor clones that secrete pro-angiogenic factors needs to devote time and energy that could be used for mitosis in a process that will benefit other tumor cells in the vicinity.

**Fig 1:**
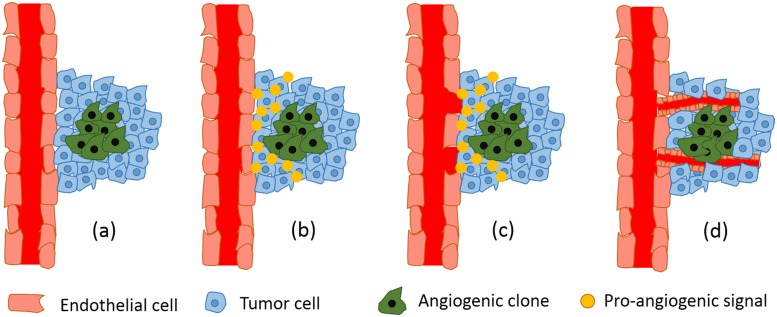
The process of angiogenesis. When tumor’s diameter excessed a critical range (2 *mm*) (a), angiogenic clones start to secrete pro-angiogenic signals (b). The signals degrade capillary vessel wall (c) and finally results in migration of endothelial cells and formation new tubes with a central lumen (d).

Angiogenesis is the process whereby new blood vessels are formed from extant vessels. It has a vital role in supplying nutrients and oxygen, and in driving out metabolic waste products [6]. Lack of vascular support can limit tumor growth and lead to the formation of necrotic cores [7]. Hypoxia has been thought as a primary regulator of angiogenesis: in a hypoxic tumor, angiogenic clones promote factors such as hypoxia inducible factor 1 (HIF-1), tumor angiogenic factors (TAF), in particular vascular endothelial growth factor (VEGF) [8], and angiopoietin-2 (ANG2) [9]. Today, it has been accepted that the angiogenic switch, is regulated by the balance between proangiogenic and antiangiogenic factors in the solid tumor microenvironment [9–11]. Whenever pro-angiogenic factors are more than anti-angiogenic factors, they may induce the switch to an angiogenic phenotype [9]. Based on currently available data, hypoxia produces pro-angiogenic factors such as VEGF, IL-8, angiogenin, FGF and PDGF and anti-angiogenic such as DLL1-4, Vasohibin-1, Interferons (IFN-alpha, IFN-beta, IFN-gamma) [12, 13]. These factors induce nearby vascular endothelial cells to proliferate. Then daughter endothelial cells form new blood vessels following the VEGF concentration gradient [14]. Fig 1 shows the angiogenesis process steps. Note that there are several molecular mechanisms to induce angiogenesis in solid tumors that are out of the scope of this paper.

The process of angiogenesis has received a substantial amount of attention from experimental cancer researchers as well as mathematical modelers [15]. Angiogenic clone (clone that secretes pro-angiogenic factors) invests energy and resources that could be put into proliferation to secrete factors that will help it but also other cells in the tumor. As well as nutrient delivery and waste removal, angiogenesis provides tumor micro-vessels that facilitate metastasis. Hence, tumor cells gain the advantages of angiogenesis irrespective of whether they contribute to produce angiogenic factors or not. This makes pro-angiogenic factors a public good which could be vulnerable to exploitation by free-riders (clones that decrease or cease their own contribution in producing pro-angiogenic signals). Given their lower contribution to the public good and the energetic savings that result from that, natural selection should favor the free-riding clones. But the reason tumors grow and become aggressive and lethal to the patient suggests that angiogenic cancer cells are not sufficiently eradicated from the tumor population.

Here, the evolutionary problem is why angiogenic clone is not dominated by other mutant clones (e.g., proliferative clone). This situation can be framed in terms of game theory as a prisoner’s dilemma where angiogenic and free-rider clones could be thought as cooperator and defector, respectively.

One of the most important element of cancer progression and development is the secretion of diffusible factors (e.g. growth factors) by the tumor clones [16]. Clones within tumor can promote each other’s growth by producing the factors. Clones that don’t produce growth factors can also sustain their replication power using the diffused factors from neighboring cells. Hence, secretion of diffusible factors by tumor clones is a type of cooperation among cancer cells. Scientists have demonstrated that tumor clones cooperate for the secretion of growth factors, that is, cells that do not secrete the growth factor can benefit from the products of their neighbors [17, 18]. In this cooperation, the diffusible factors are public goods [19, 20], because their effect is not limited to the producer cell. Therefore evolutionary dynamics of tumor can be studied in the framework of Public Goods Game where clone’s fitness are determined by collective interactions. So far the PGG has been used to model the interactions of tumor clones in several research work, such as [19, 20, 20–23]. In this study, we will model angiogenic factors (i.e., public goods) and the emergence and maintenance of angiogenic clone in the tumor population as a structured Public Goods Game (PGG).

In a PGG, all individuals have the opportunity to invest an amount *c* into a common pool. The total amount is then multiplied by a positive number *r*, and is distributed equally among all individuals, regardless of whether they have contributed or not. The fitness for cooperators and defectors among N players in an infinite population clone are given by:

WC=∑j=0N-1N-1Jxj(1-x)N-j-1(j+1)cN-c (1)

WD=∑j=0N-1N-1Jxj(1-x)N-j-1jcN (2)

where *x* is the relative frequency of the cooperation in the population. In this configuration, every individual has a temptation to defect since the cost of defection is equal to zero. Thus, each rational player avoids contributing and makes an effort to free ride on contributions of other players. If everybody follows this rationality, no one would contribute and hence the benefits of the public good would be equal to zero.

There are many evidences that demonstrate spatial effects can influence the evolutionary dynamics of the public goods game [22, 24, 25]. More precisely, diffusing the public goods (e.g., pro-angiogenic signals) are influenced by the structure of the population that impact it through space [26–30]. Hence, spatial structure can promote ar inhibit evolution of cooperation in population. This problem has extensively studied in evolutionary pairwise game but the evolution of public goods on structured population make known a new phenomenon that must be modeled.

### Related work

So far, several game theoretical models have been proposed to investigate angiogenesis process [31–35]. Some of them have assumed that pro-angiogenic clone influences one cell in the neighbors (i.e., pairwise interaction) [31, 33, 33]. This assumption, however, is unrealistic. In reality, clearly pro-angiogenic clone and factors have a collective effect on a large number of cells, not just one cell engaged in a pairwise interaction. Nagy has presented a system of nonlinear ordinary differential equations tracking the mass of two different cell populations in the tumor and the mass of vascular endothelial cells [34]. He has investigated that how the mutant phenotype in tumor can dominate in the population. The model includes an energy management system. According to his model natural selection always selects the highest proliferative clone, but is neutral about pro-angiogenic clone that secrets growth factors. According to his model, pro-angiogenic clone to spread in the population should be more proliferative than other clones within tumor. But, we know that, ATP that is allocated to secretion growth factor necessarily decreases ATP available for proliferation, hence according to his model natural selection will disfavor pro-angiogenic clone, because this clone have less energy to more proliferation. Moreover, this model don’t investigate costs associated with hallmark phenotypes that leads to misleading [35, 36]. This limitation has been addressed in a new study by Nagy and Armbruster [36] in which the previous model [34] has been extended to include “cost”.

Nagy and Armbruster in the new study have investigated the evolution of pro-angiogenic and proliferative traits in tumors by extending the previous work [36]. They added costs to dynamics of proliferation and angiogenesis strategies in the form of energy requirements. In this model, natural selection decides solely on the proliferative potential, similar to the findings of previous study [34]. Nonetheless, in this model natural selection can select mutations that increase pro-angiogenic factors, but because of pleiotropic effect (i.e., obtaining more ATP) not because of their abilities for producing pro-angiogenic factors. In this way, free-riders cannot benefit by abstaining pro-angiogenic secretion since by doing so they decrease ATP available for proliferation.

In other work, Bickel et al. have presented a model to describe angiogenesis [35]. They have presented a stochastic simulation based on the evolutionary model by Nagy and Armbruster [36]. The only alteration that they applied is at the evolutionary scale. Moreover, in their model, the population can include indefinite number of clones to interact simultaneously, in addition to the mutant clone that arises at random times. They have relaxed the adaptive dynamics assumptions and have explained that they have no significant influences on evolutionary endpoints rather than the original adaptive dynamics.

These three studies [34, 34, 35], have modeled tumor-stroma interactions. Based on these studies pro-angiogenic clone can spread in the population, but not because of direct selection on pro-angiogenic factor secretion. Pro-angiogenic clone is favored by natural selection only as a pleiotropic effect (i.e., obtaining more ATP). In contrast with these three studies, we have modeled the interactions of two Pro and anti-angiogenic clones within a tumor. We have shown that pro-angiogenic clone can evolve and spread in the population because of direct selection on spatial secretion of pro-angiogenic factors (not because of considering pleiotropic effect). Moreover, in contrast with the previous studies [34, 34, 35], we have shown, due to frequency-dependent selection, the coexistence of pro and anti-angigenic clones are possible.

In different studies, Archetti has presented two models to investigate producer and non-producer phenotypes in the public goods game [19, 37]. In the first model, Archetti has proposed a public goods game for growth factor production [19]. He has shown direct selection on growth factor secretion can result in five types of dynamics (including the coexistence of producer and non-producer cells). Archetti in the new study has investigated the spatial effect on the public goods game by extending the previous work [37]. He shows that nonlinear benefit function can result in coexistence of two phenotypes on the lattice. However, in this study, unlike the Archetti’s model, we have assumed that each player on any given graph participates in several public goods games, simultaneously. Our results, in contrast with Archetti’s model, demonstrate that cooperation phenotype continues to exist even by linear payoff.

## Materials and methods

### Evolution of angiogenesis by spatial public goods game

**Fig 2:**
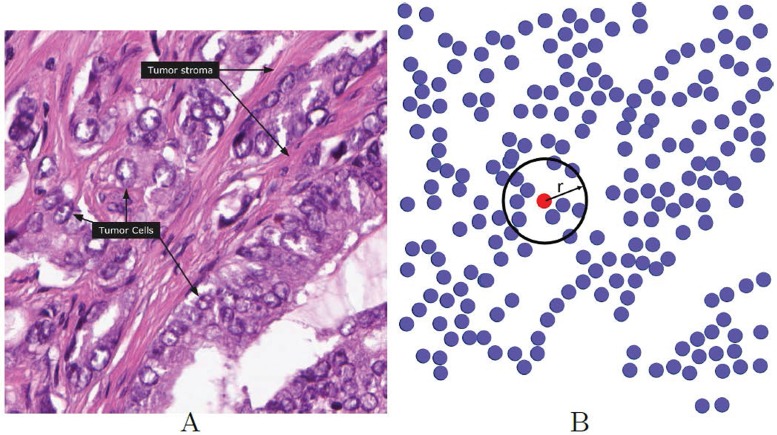
A sample of human-prostate cancer tissue seen from the proteinatlas station (http://www.proteinatlas.org/learn/dictionary/cancer/prostate+cancer+3). Panel B indicates tumor cells that have been shown in panel A. As shown, each tumor cell interacts with all neighbors living in a distance *r* from this cell, by pro-angiogenic factors diffusion.

Most cancers are solid masses of tissue except some that are related to blood and bone marrow. Structure of solid neoplasm is shaped by the normal tissues [38]. For instance, malignant prostate cancer, mostly arises from epithelial cells in the peripheral zone. In a monolayer epithelium, prostate epithelial cells have a shape resembling that of a polygon. These polygons juxtapose to compose a tiled array free from gaps (please see Fig 2). This topology can be represented by a graph in which each node represents a tumor cell. In our model we will assume that cells that are linked share pro-angiogenic factors [39].

**Fig 3:**
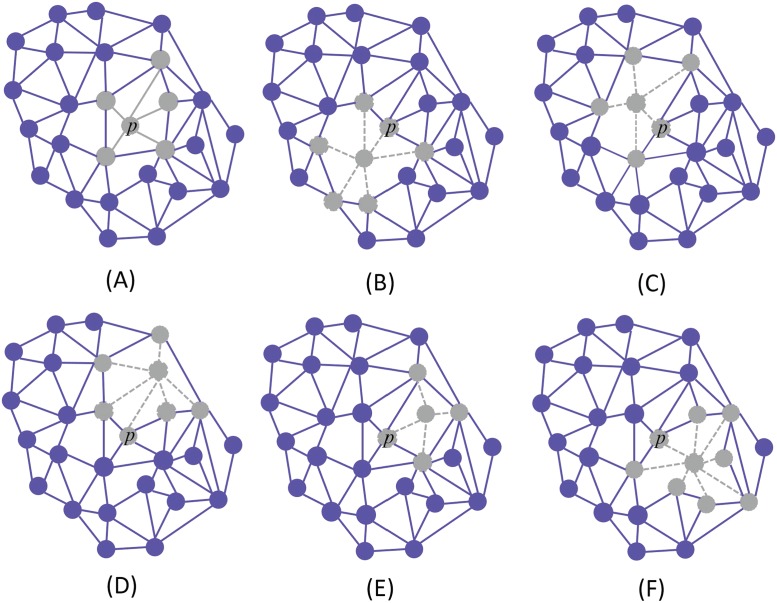
Each cell on the graph plays *k* + 1 PGGs with *k* neighbors. Node *p* participates in six PGGs, simultaneously. Panels (A)-(F) show all these games.

Using this graph organization we will now consider evolutionary dynamics on the graph using public goods game. In our problem, the graph has two types of nodes: 1) pro-angigenic clone (that produces pro-angigenic factors) 2) free-rider clone (that don’t contribute to angiogenesis). Each cell participates in multiple public good games with the neighbors. For example, in Fig 3A, node *p* engages in a PGG along with its five neighbors (the group size of this PGG is 6). However, the focal cell participates in several PGGs; that centered on its neighborhood *p* plus those connected with the neighborhoods centered on the focal cell neighbors. Panels B-F of Fig 3 indicate other PGGs that *p* participates in. In these games the focal cell is *p*’s neighbors. Hence, a focal cell with *k* neighbors will engage in *k* + 1 PGGs. Note that graph heterogeneity results in that the cells participate in different numbers of PGGs.

Each cell can contribute different *c* in each PGG. However, in this paper a cell contributes $\frac{c}{k+1}$ in each PGG (where *c* is total contribution for the focal cell and *k* is the number of the focal cell’s neighbors). That is, the contribution equally is shared between all PGGs that the focal cell participates in them.

### Spatial linear public goods game

When the benefit function of the public goods is linear, payoff of a focal cell who decides to play the games on a graph as a cooperator (pro-angiogenic clone), *W*_*C*_, or as a defector (free-rider clone), *W*_*D*_, are given by, respectively:

WC(x)=∑i=0kf∑j=0kni(f)kni(f)jxj(1-x)kni(f)-j(cknj(ni(f))+1)(r(j+1)kni(f)+1)-c (3)

WD(x)=∑i=0kf∑j=0kni(f)kni(f)jxj(1-x)kni(f)-j(cknj(ni(f))+1)(rjkni(f)+1) (4)

where *f* is the focal cell, *k*_*f*_ is the number of neighbors of cell *f*, and *n*_*i*_(*f*) indicates *i*^*th*^ neighbor of cell *f*. In a clonal population, the replicator dynamics [40] of this game is given by:

$$\begin{array}{c} \dot{x}=x\left(1-x\right)\left(W_{C}\left(x\right)-W_{D}\left(x\right)\right) \end{array}$$ (5)

So, we have

x˙=x(1-x)(∑i=0kf∑j=0kni(f)kni(f)jxj(1-x)kni(f)-j(rc(knj(ni(f))+1)(kni(f)+1)-c) (6)

If the graph has vertices of degree *k* (i.e., *k*-regular graph), the replicator dynamics will be:

$$\begin{array}{c} \dot{x}=x\left(1-x\right)\left(\frac{rc}{k+1}-c\right) \end{array}$$ (7)

### Spatial nonlinear public goods game

**Fig 4:**
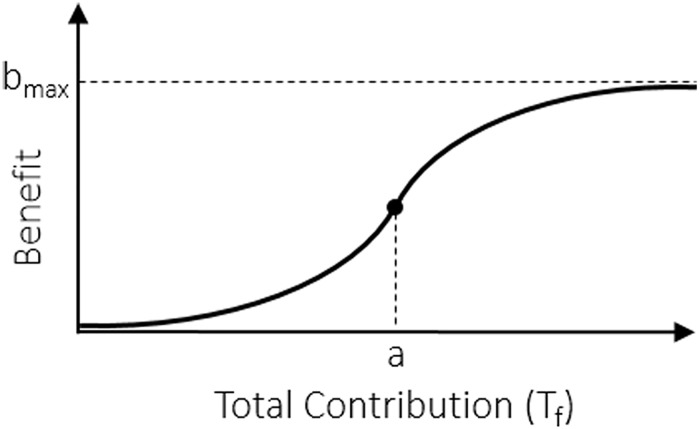
Benefit function that has sigmoid shape. The function displays a progression with small beginnings that followed by dramatical growth. In this figure, *a* shows the inflection point of the function and *b*_*max*_ represents the maximum benefit that an individual can obtain.

So far, we have assumed that the benefit of the public good is a linear function of the number of cooperators. However, a large variety of natural phenomena are not linear and are more likely to resemble logistic behavior [41–43]. Benefit function in tumor angiogenesis, obviously, has such behavior. When the pro-angiogenic factors (i.e., contributions by the all local cooperator cells) are low, a new vascular infrastructure isn’t induced and consequently the benefit will be near to zero. By increasing the contributions, the benefit accelerates and approaches to its maximum value (Fig 4).

There are several sigmoid functions such as: logistic, hyperbolic tangent, algebraic, Gauss error, and Hill functions. We use algebraic form to represent the benefit function in Fig 4. So, the benefit is given by:

$$\begin{array}{c} b\left(T_{f}\right)=\frac{b_{max}}{2}+\frac{T_{f}-a}{\sqrt{s+{\left(T_{f}-a\right)}^{2}}}\times \frac{b_{max}}{2} \end{array}$$ (8)

where *s* is the slope of the curve at inflection point *a* (with $0<a\leq \frac{1}{2}c$ and *s* > 0). *T*_*f*_ shows the total contribution by *f* and neighbors, that in our problem is given by Eqs 9 or 10 based on the type of the focal player *f*.

TfC=∑i=0kf∑j=0kni(f)kni(f)jxj(1-x)kni(f)-jc(j+1)(knj(ni(f))+1)(kni(f)+1) (9)

TfD=∑i=0kf∑j=0kni(f)kni(f)jxj(1-x)kni(f)-jcj(knj(ni(f))+1)(kni(f)+1) (10)

where *f*_*C*_ (*f*_*D*_) represents the focal individual that decides to cooperate (defect). The replicator dynamics [40, 44] of this game are given by:

$$\begin{array}{c} \dot{x}=x\left(1-x\right)\left(b\left(T_{f_{C}}\right)-c-b\left(T_{f_{D}}\right)\right) \end{array}$$ (11)

For the *k*-regular graph, the replicator dynamics will be:

$$\begin{array}{c} \dot{x}=x\left(1-x\right)\left(\frac{2c(k(x-a)+1)}{\sqrt{s{\left(k+1\right)}^{2}+c^{2}{\left(k\left(x-a\right)+1\right)}^{2}}}-\frac{2ck(x-a)}{\sqrt{s{\left(k+1\right)}^{2}+c^{2}k^{2}{\left(x-a\right)}^{2})}}-c\right) \end{array}$$ (12)

where *b*_*max*_ = 4.

## Results

In the previous section, we proposed two games with linear and nonlinear benefits. In the next, we investigate the evolutionary dynamics of the pro-angiogenic and free-rider clones and the stability in the games.

### Angiogenesis with linear benefit

**Fig 5:**
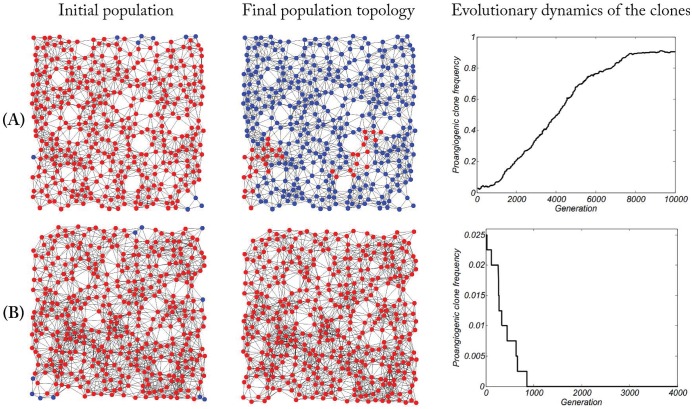
Three examples for evolution of cooperation on irregular graph (blue and red nodes show pro-angiogenic and free-rider clones, respectively). Panel (A) represents an examples of spreading pro-angiogenic clone in the population. In this example, coexistence between pro-angiogenic and free-rider clones arises over time. While panel (B) demonstrates how pro-angiogenesis clone can go extinct in a dense graph. In this example, free-riders lonely spread in the population and act as a stable strategy. We assume that all nodes in the graph have free-rider strategy (red nodes) and then a small fraction of them are mutated to pro-angiogenic clone (blue nodes). Left column shows initial populations including the mutant nodes, middle column represents final topology that has been shaped by natural selection forces over time, and right column indicates fraction of pro-angiogenic clone in the population over time. As shown, both free-rider and pro-angiogenic clones can coexist on irregular graphs. Simulation codes are available in S1 File.

In the game with linear benefit, the evolutionary dynamics on the regular graph have two trial fixed points *x* = 0 and *x* = 1. Each of them can be stable in the population based on the value of $\frac{r}{k+1}$. If $\frac{r}{k+1}>1$ natural selection favors pro-angiogenic mutants, otherwise free-riders can be selected. Hence, on a regular graph, pro-angiogenic and free-rider clones cannot be in a coexistence game. However, our simulations show that coexistence between the clones can arise on irregular graph. Fig 5(A) demonstrates this evidence. In the simulations, cells (i.e., anti-angiogenic clone) are randomly distributed in an area with a given density which is constant over time. Each cell interacts with its neighbors. In each generation, some cells are randomly selected and the new payoffs are calculated according to the interactions with the neighbors (please consider Eqs 3 and 4). Then the strategy of the selected cells will be updated proportional to the neighbor payoffs. This update model indicates the fact that the cells want to take the strategy of the neighbor with greater fitness. End points in our simulation are asymptotic that are associated to the tumor’s structure and initial mutant positions. These points are not long-term transient behavior, because in each step of our long time simulation, several mutations occur that can change the final states. Hence, if the points are long time transient, they most likely changed through mutations in the primary generations.

By increasing *k*, where it shows the density of the graph, free-riders can lonely spread in the population and act as a stable strategy. Fig 5(B) shows a dense graph in which the mutants (pro-angiogenic clone) go to extinct finally.

### Angiogenesis with nonlinear benefit

In the game with nonlinear benefit (Eq 12) the evolutionary dynamics have some interior fixed points that are given by the roots of $\frac{2c(k(x-a)+1)}{\sqrt{s{\left(k+1\right)}^{2}+c^{2}{\left(k\left(x-a\right)+1\right)}^{2}}}-\frac{2ck(x-a)}{\sqrt{s{\left(k+1\right)}^{2}+c^{2}k^{2}{\left(x-a\right)}^{2})}}-c$, in addition to two trivial fixed points *x* = 0 and *x* = 1.

**Fig 6:**
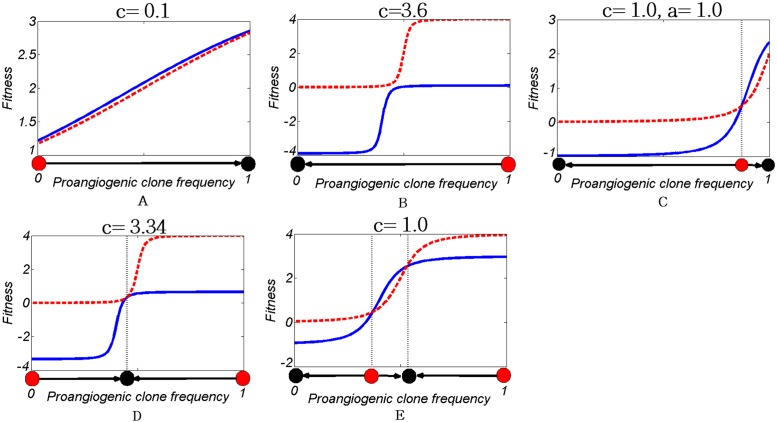
Types of evolutionary dynamics with regards to the model’s parameters. Each panel plots the fitness of two clones based on the relative frequency of the pro-angiogenic. The meeting point of two fitness indicates stable equilibrium of the game (red and black circles show unstable and stable points of the game in each panel). The game has five types of the evolutionary dynamics. 1- Panel A: only pro-angiogenic clone (*x* = 1) is stable 2- Panel B: only free-rider clone (*x* = 0) is stable 3- Panel C: both pro-angiogenic and free-rider clones are stable 4- Panel D: only a polymorphic population (*x* = *x*_*s*_ where 0 ≤ *x*_*s*_ ≤ 1) is is stable 5- Panel E: both polymorphic population and pro-angiogenic clone are stable.

Let’s assume that *c* = 1, *k* = 10, *a* = 0.5, *s* = 0.01, and *b*_*max*_ = 4, therefore the fixed points will be *x* = 0, *x* = 1, *x* = 0.369, and *x* = 0.530. The dynamics (Eq 12) with regard to the model parameters (i.e., *c*, *b*_*max*_, *s*, *a*, and *k*), potentially, have 5 types of evolutionary dynamics (Fig 6).

**Fig 7:**
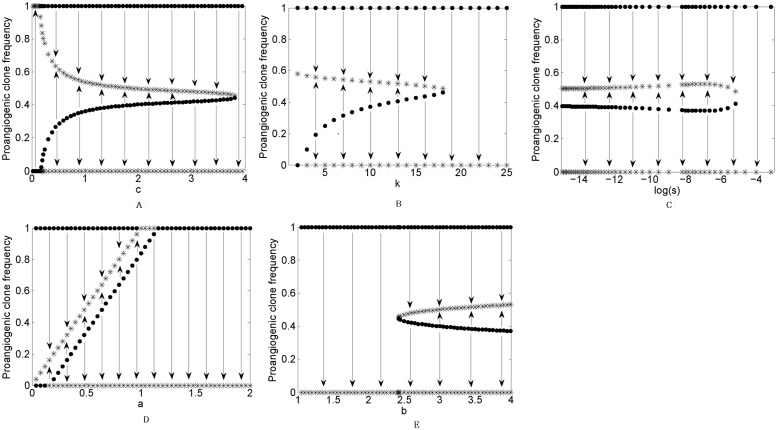
Bifurcations in heterogeneous populations of pro-angiogenic and free-rider clones interacting in the public goods game with initial values *k* = 10, *c* = 1, *b* = 4, *s* = 0.01, *a* = 0.5. The model parameters has been considered as the bifurcation parameter in the plots. Stars and circles in the plots demonstrate stable and unstable points in the system. Arrows show the direction of population evolutionary dynamics.

Obviously, for low *c*, angiogenic switch occurs (i.e., pro-angiogenic clone dominates the population). Increasing the contribution *c* results in blocked angiogenic switch. The bifurcation diagram in Fig 7 represents the effect of the parameters on the stability of system’s equilibria. As shown in this figure, nonlinear benefit can induce polymorphism, i.e., coexistence of two stable strategy simultaneously.

The types of the system dynamics are depend on: *(i)* the slope of the benefit curve (*s*) at the inflection point *a*, and *(ii)* the fraction of pro-angiogenic factors that is necessary before angiogenesis (*T*_*f*_). Decreasing the slope triggers angiogenic switch, while increasing total pro-angiogenic factors up to a threshold in the environment induces angiogenic switch. The plot shows that plenty more pro-angiogenic factors block angiogenic switch. As expected, by increasing the size of the neighborhood, the cells move from the strong pro-angiogenesis state to blocked angiogenesis, because the PGG convert to pairwise prisoner’s dilemma.

**Fig 8:**
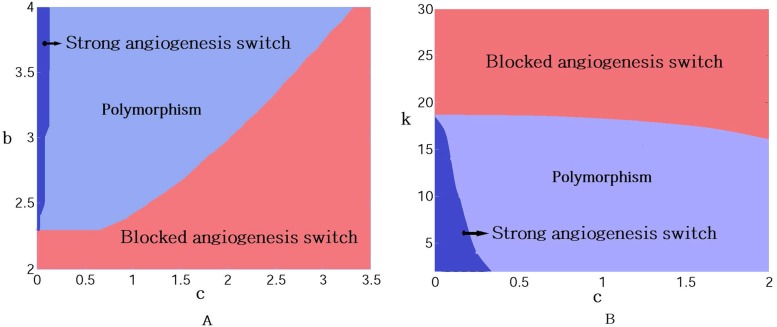
Characterization of different levels of angiogenic switch in the parameter space of *c* and *b* (right panel), and *c*, *k* (left panel). The red region corresponds to cells that are free-riders (weak angiogenesis), the dark blue region demonstrates cells that strongly produce pro-angiogenic factors (strong angiogenesis), and the pale blue represents a mixed stable strategy between pro-angiogenic and free-rider clones.

There are critical values for the model parameters that determine the types of evolutionary dynamics. These values are not independent. This leads to different basin of attractions in the model parameters space. Fig 8 shows different basin of strong and blocked angiogenesises based on the different values of *c*, *b*, and *k*. Only for the small enough contribution *c*, pro-angiogenic clone lonely invades tumor.

## Discussion

Heterogeneity is one of the driving forces in evolution. Most mathematical models based on evolutionary game theory assume well-mixed populations where individuals interact with each other with a probability given by their respective proportions in the population. This assumption simplifies mathematical analysis but obscures the important role of population structure (i.e. spatial heterogeneity) in the interactions between different cancer clones in a growing tumor. The emergence of pro-angiogenic clones in solid tumors is one of these scenarios in which structure and spatial effects are determinant. The emergence of pro-angiogenic clones, a critical hallmark in cancer, seems counter-intuitive if we assume a parental population made of non-cooperative clones (with regards to angiogenesis). According to the results, it might be concluded spatial heterogeneity in the tumors is one of the significant elements to drive the evolutionary dynamics involved in the emergence of angiogenic clones. The results also showed that the possibility of coexistence of pro-angiogenic and free-rider clones, when the benefits of pro-angiogenic factors are nonlinear. The simulation results demonstrate denser graphs decrease the probability of coexistence compared to less sparse ones. In other words, by increasing the number of neighbors of each cell the population will be inviscid, the proportion of free-riders increases and the angiogenesis are made less viable. The simulation results show the position of initial mutant cells has a critical role in the evolutionary dynamics process. We have investigated linear and nonlinear benefits on the regular and irregular structures (i.e., graphs). Our findings demonstrate that the linear benefit may only induce coexistence on the irregular graphs.

**Fig 9:**
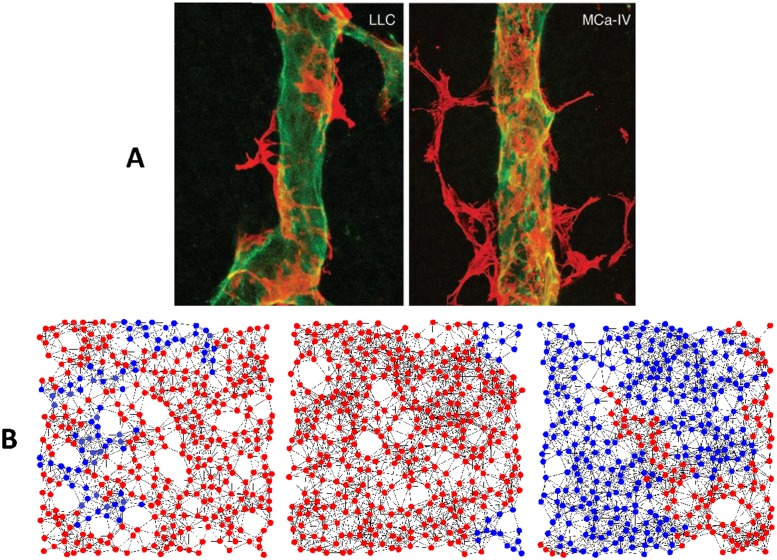
Configuration of pro-angigenic mutants in the surrounding vascular neoplasm. Pro-angiogenic clone in tumors equilibrates into one or several clusters that begin from surrounding vascular tissues and go towards the center of tumor. A: pro-angigenic mutants for Lewis Lung Carcinoma (LLC) and MCa-IV mammary carcinoma. Red and green colors indicates pro-angiogenic clone and blood vessels in the tumor [Panel A from Morikawa et al. [45], with permission]. B: Configuration of pro-angigenic clone in our simulations. The mutant cells (blue node) equilibrate into one or several randomly composed cluster, without considering the size and shape of the clusters and mutants. The graphs demonstrates final equilibrium configurations over the evolutionary process. Simulation codes are available in S1 File.

Furthermore, our simulations show that the pro-angiogenic mutant in the population equilibrates into several clusters, even though the initial mutants have been scattered throughout the population. In other words, there is a critical mass of cooperators necessary for pro-angiogenic clone to induce angiogenesis and to be self-sustaining. Moreover, the simulation results demonstrate that the sprouting of new vessels starts from surrounding vascular tissues towards the center of tumor. Fig 9 shows these phenomena for Lewis lung carcinoma (LLC) and MCa-IV mammary carcinoma and in our simulations that are in accordance with [45–47]. This observation is completely according to the nature of angiogenesis. It should be noted that we don’t aim to claim our graphs in this figure are completely in accordance with real situations. However, we only want to represent a biological fact that can be captured by the model: tumor vessels have a tendency to violate all the conventional rules of microvasculature, developing without any organization, following tortuous paths and changing in diameter without any configuration [48]. Daughter endothelial cells form new blood vessels following the pro-angiogenic factors concentration gradient; if the concentration is low the pro-angiogenic factors cannot induce the nearby vascular endothelial cell to proliferates. Therefore, pro-angiogenic factors should be increased up to a threshold in a local area to from new vessels. This process is done by forming a combination of the separated pro-angiogenic clones into a cluster. Note that our graphs in this figure show pro and anti-angiogenic clone rather vessels.

**Fig 10:**
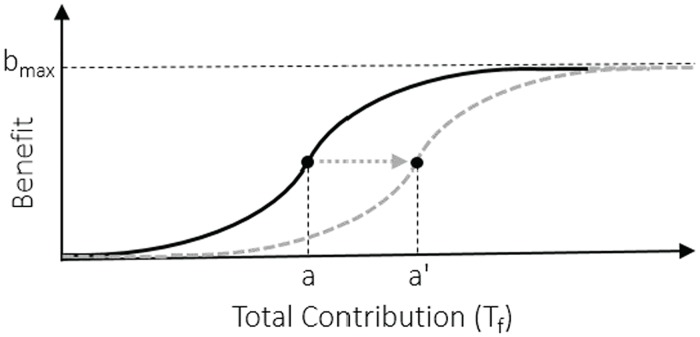
Anti-angiogenesis therapy changes the benefit function. In fact, anti-angiogenesis therapy can change *b*_*max*_, *s*, and inflection point *a*. In this figure inflection point *a* has been changed to *a*′. Dashed curve shows new benefit function.

**Fig 11:**
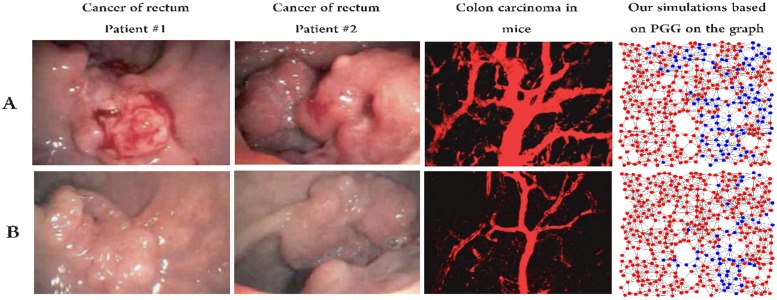
Anti-angiogenesis therapy can restore tumor vessels towards a more normal behavior. In this figure, there are several examples of tumors that have became dramatically less hyperemic after treatment. These examples (from left to right) are from Willett et al. [51] and Jain [52], respectively, with permission. A: Tumors before tratment. B: Tumor after anti-angiogenesis treatment. As shown in the right panels, our simulations can predict such normal state. In our simulations, after binding inhibitors to the receptors, the number of pro-angiogenic clone that are representative of the tumor blood vessels decreases. Simulation codes are available in S1 File.

Binding of pro-angiogenic factors to their receptors trigger the signaling cascade that controls and maintains endothelial cell mitogenesis and migration and consequently induces the sprouting of new vessels. Hence, pro-angiogenic factors and their receptors are the main target of signaling pathway in angiogenesis. All these pathways cooperate to angiogenesis synergistically. Anti-angiogenic therapies affect the pathways by blocking certain specific receptors. However, none of them completely inhibit all the components of pro-angiogenic signaling, and therefore the sprouting of new vessels may continue through the other pathways. For example, bevacizumab, a licensed angiogenesis inhibitors, recognizes and attaches to VEGF-A (a VEGF receptor). Whenever VEGF-A is binded to bevacizumab, VEGF is unable to trigger the VEGF receptor and it is sequestered. Therefore, the inhibitors that block the receptors transform the benefit function (please see Fig 10). Essentially, anti-angiogenic therapies recover the balance between pro-angiogenic and other factors within tumors by changing the benefit function of the involved agents. This, at least temporarily, derive the tumor vessels toward a more regular behavior. Angiogenic inhibitors have been known to affect different stages of angiogenesis. The most effectiveness of these inhibitors is reducing the volume of tumor vessels and restoring them to a more normal behavior [49, 50]. This role of the anti-angiogenic agents has observed in several tumors [45, 47]. Our simulations justify this behavior of the inhibitors (please see Fig 11). It should be noted that our model is qualitative rather than quantitative. Hence, it shows only the general behavior of tumors under anti-angiogenic treatment. Note that, whenever cancer cell migration and growth is slower than the inhibitors dynamics within the tumors, then it develop resistance to the anti-agiogenic therapy over time, after a couple of months of the treatment. We will further investigate this aspect in future research.

## Conclusion

Our study has shown a mathematical formalism using structured evolutionary public goods game to demonstrate that why angiogenic clone can invade the population despite it has cooperating trait that is susceptible to free-riding. The proposed framework can be used to model other populations with the same specifications. Although in this paper we have modeled the phenotypes that secret pro-angiogenic factors or free rid, other biological candidate molecules in populations of cancer cells can be considered. The results of the proposed model demonstrate the coexistence of two stable states (polymorphism) in tumor ecology in most cases. The evolutionary dynamics on graph demonstrated that a simple pro-angiogenic mutation in some tumor microenvironments can lead to a critical transition between free-riding and angiogenesis traits. Generally speaking, the results showed by employing a multi-player game and considering the structure of tumor as a critical factor, we can demonstrate why the angiogenesis trait can coexists with other clones in malignant tumors. Our results demonstrated that pro-angiogenic clone equilibrates into clusters that appear from surrounding vascular tissues towards the center of tumor. These clusters appear notably less dense after anti-angiogenic therapy. Also, in this paper we investigated the effect of contribution, size of neighbors, and the type of benefit function on the evolutionary dynamics of pro-angiogenesis mutants.

## Supporting information

S1 File The simulation codes for Figs 5, 9 and 11. (ZIP)
